# Management in severe dementia: recommendations of the Scientific Department of Cognitive Neurology and Aging of the Brazilian Academy of Neurology

**DOI:** 10.1590/1980-5764-DN-2022-S107PT

**Published:** 2022-11-28

**Authors:** Sonia Maria Dozzi Brucki, Ivan Aprahamian, Wyllians Vendramini Borelli, Victor Calil da Silveira, Ceres Eloah de Lucena Ferretti, Jerusa Smid, Breno José Alencar Pires Barbosa, Lucas Porcello Schilling, Márcio Luiz Figueiredo Balthazar, Norberto Anízio Ferreira Frota, Leonardo Cruz de Souza, Francisco Assis Carvalho Vale, Paulo Caramelli, Paulo Henrique Ferreira Bertolucci, Márcia Lorena Fagundes Chaves, Ricardo Nitrini, Rodrigo Rizek Schultz, Lilian Schafirovits Morillo

**Affiliations:** 1 Universidade de São Paulo, Faculdade de Medicina, Departamento de Neurologia, Grupo de Neurologia Cognitiva e do Comportamento, São Paulo SP, Brazil.; 2 Faculdade de Medicina de Jundiaí, Departamento de Medicina Interna, Divisão de Geriatria, Grupo de Investigação sobre Multimorbidade e Saúde Mental no Envelhecimento, Jundiaí SP, Brasil.; 3 University of Groningen, University Medical Center Groningen, Department of Psychiatry, Groningen, The Netherlands.; 4 Universidade de São Paulo, Faculdade de Medicina, Hospital das Clínicas, Serviço de Geriatria, Laboratorio de Investigação Médica em Envelhecimento, São Paulo SP, Brasil.; 5 Hospital de Clínicas de Porto Alegre, Departamento de Neurologia, Porto Alegre RS, Brasil.; 6 Instituto D’Or de Pesquisa e Ensino, Rio de Janeiro RJ, Brasil.; 7 Hospital Glória D’Or, Rio de Janeiro RJ, Brasil.; 8 Universidade Federal de Pernambuco, Centro de Ciências Médicas, Área Acadêmica de Neuropsiquiatria, Recife PE, Brasil.; 9 Instituto de Medicina Integral Prof. Fernando Figueira, Recife PE, Brasil.; 10 Pontifícia Universidade do Rio Grande do Sul, Escola de Medicina, Serviço de Neurologia, Porto Alegre RS, Brasil.; 11 Pontifícia Universidade do Rio Grande do Sul, Instituto do Cérebro do Rio Grande do Sul, Porto Alegre RS, Brasil.; 12 Pontifícia Universidade do Rio Grande do Sul, Programa de Pós-Graduação em Gerontologia Biomédica, Porto Alegre RS, Brasil.; 13 Universidade Estadual de Campinas, Faculdade de Ciências Médicas, Departamento de Neurologia, Campinas SP, Brasil.; 14 Hospital Geral de Fortaleza, Serviço de Neurologia, Fortaleza CE, Brasil.; 15 Universidade de Fortaleza, Fortaleza CE, Brasil.; 16 Universidade Federal de Minas Gerais, Departamento de Clínica Médica, Belo Horizonte MG, Brasil.; 17 Universidade Federal de São Carlos, Centro de Ciências Biológicas e da Saúde, Departamento de Medicina, São Carlos SP, Brasil.; 18 Universidade Federal de São Paulo, Escola Paulista de Medicina, Departamento de Neurologia e Neurocirurgia, São Paulo SP, Brasil.; 19 Hospital de Clínicas de Porto Alegre, Serviço de Neurologia, Porto Alegre RS, Brasil.; 20 Universidade Federal do Rio Grande do Sul, Faculdade de Medicina, Departamento de Medicina Interna, Porto Alegre RS, Brasil.; 21 Associação Brasileira de Alzheimer, São Paulo SP, Brasil.; 22 Universidade Santo Amaro, Departamento de Neurologia, São Paulo SP, Brasil.; 23 Universidade de São Paulo, Hospital das Clínicas, Serviço de Geriatria, São Paulo SP, Brasil.

**Keywords:** Dementia, Palliative Care, Behavior, Cognition, Demência, Cuidados Paliativos, Comportamento, Cognição

## Abstract

Alzheimer’s disease (AD) and other neurodegenerative dementias have a progressive course, impairing cognition, functional capacity, and behavior. Most studies have focused on AD. Severe dementia is associated with increased age, higher morbidity-mortality, and rising costs of care. It is fundamental to recognize that severe dementia is the longest period of progression, with patients living for many years in this stage. It is the most heterogeneous phase in the process, with different abilities and life expectancies. This practice guideline focuses on severe dementia to improve management and care in this stage of dementia. As it is a long period in the continuum of dementia, clinical practice should consider non-pharmacological and pharmacological approaches. Multidisciplinary interventions (physical therapy, speech therapy, nutrition, nursing, and others) are essential, besides educational and support to caregivers.

## RESUMO

A doença de Alzheimer (DA) e outras demências neurodegenerativas têm um curso progressivo com comprometimento da cognição, capacidade funcional e comportamento. A maioria dos estudos enfocou a DA. A demência grave está associada ao aumento da idade, maior morbimortalidade e aumento dos custos de cuidados. É fundamental reconhecer que a demência grave é o período mais longo de progressão, com o paciente vivendo muitos anos nesta fase. É a fase mais heterogênea do processo, com diferentes habilidades e expectativa de vida. Esta diretriz de prática concentra-se na demência grave para melhorar o manejo e o cuidado nessa fase da demência. Como um longo período no continuum da demência, as abordagens não farmacológicas e farmacológicas devem ser consideradas. Intervenções multidisciplinares (fisioterapia, fonoaudiologia, nutrição, enfermagem, entre outras) são essenciais, além de educacionais e de apoio aos cuidadores.

## INTRODUCTION

A lzheimer’s disease (AD) and other neurodegenerative dementias have a progressive course, impairing cognition, functional capacity, and behavior. Most studies have focused on AD. Severe dementia is associated with increased age, higher morbidity-mortality, and rising costs of care. Severe stages could account for 70 to 80% of total treatment expenses^1^ ^),(^ ^2^. Degenerative dementias slowly and progressively worsen. In its severe stage, patients show a higher dependency for all basic activities of daily living and incapacity for instrumental activities of daily living.

Around 30 to 60% of patients with dementia (PWD) and 90% of individuals residing in long-term care facilities are in its moderate-late stage^3^ ^)-(^ ^5^.

The progression of dementia is associated with a progressive dependency on caregivers, with loss of capacity to provide self-care in basic activities of daily living. Usually, severe AD patients will score below ten on mini mental examination (MMSE) and moderately severely with 10 to 15^5^. Severe dementia could cause complications such as immobility, swallowing disorders, malnutrition, and fragility. This situation can increase the risk of pneumonia, which has been found as a common cause of death in PWD^5^ ^)-(^ ^9^.

It is fundamental to recognize that severe dementia is the longest period of progression, with patients living for many years in this stage. It is the most heterogeneous phase in the process, with different abilities and life expectancies^7^. Around 17% of PWD older than 75 years are living with a very severe stage of dementia^8^.

This practice guideline focuses on severe dementia to improve management and care in this stage of the disease. As a long period in the continuum of dementia, clinical practice should consider non-pharmacological and pharmacological approaches. Multidisciplinary interventions (physical therapy, speech therapy, nutrition, nursing, and others) are essential, besides educational and support to caregivers. Primary, secondary, and tertiary care center professionals could use this practice guideline .

## STAGING SEVERE DEMENTIA

We find many different situations and stages of severe dementia. A bedridden patient with dysphagia is different from a walking and dependent but communicative and without dysphagia patient.

The Clinical Dementia Rating (CDR) permits rating the severity of AD and other dementias on a five-point scale from 0 (normal) to 3 (severe stage)^10^. The final score is obtained after interviews with PWD and informants. It evaluates memory, orientation, judgment, problem solving, community affairs, home, hobbies, self-care. Chaves et al.^11^ ^)^ validated this study for Brazilian Portuguese.

The Alzheimer Disease Cooperative Study of activities of daily living (ADCS-ADL sev) is a good measure of activities of daily living (ADL) which strongly correlates with cognition and severity of dementia in moderate to severe AD. Its scores vary from 0 to 54 points, divided into 19 items^12^.

A simple scale that assesses gradual severity and progression of the disability during follow-up is the Global Deterioration Scale (GDS)^13^. This scale has seven global stages, from normality to severe impairment; stage 6 represents severe dementia, and stage 7, very severe^13^.

The Functional Assessment Staging (FAST) assesses decline from normal (stage 1) to severe stages (stage 7); the latter is subdivided into seven situations (A to G) (Box 1)^14^.

**Box 1:** Functional Assessment Staging (FAST).

1	No difficulty either subjectively or objectively.
2	Complains of forgetting location of objects. Subjective work difficulties.
3	Decreased job functioning evident to co-workers. Difficulty in traveling to new locations. Decreased organizational capacity.
4	Decreased ability to perform complex tasks, e.g., planning dinner for guests, handling personal finances (such as forgetting to pay bills), difficulty shopping, etc.
5	Requires assistance in choosing proper clothing.
6a	Improperly puts on clothes without assistance.
6b	Unable to properly bathe (shower) (e.g., difficulty adjusting bathwater (shower) temperature.
6c	Inability to handle toileting mechanics.
6d	Urinary incontinence.
6e	Fecal incontinence.
7a	Ability to speak limited to approximately a half a dozen intelligible different words or fewer in the course of an average day or in the course of an intensive interview.
7b	Speech ability limited to the use of a single intelligible word in an average day or in the course of an interview.
7c	Loss of ambulatory ability (unable to walk without personal assistance).
7d	Unable to sit up without assistance.
7e	Loss of ability to smile.
7f	Loss of ability to independently hold up their head.

In advanced dementia, we observe impairment to basic daily activities. The Katz Scale is often used to measure performance in bathing, dressing, toileting, transferring, continence, and feeding; characterizing severe dementia. Table 1 summarizes all these instruments.

**Table 1:** Instruments to evaluate severe dementia.

Test	Measure	Score	Answered by	Comments
SMMSE	Global cognition	0-30 points	Patient	Good for measuring cognition
CDR		0 none	Caregiver and patient	Good for follow-up
	Global State	0.5 questionable		
	Domains: memory, orientation, judgment, problem solving, community affairs, home and hobbies, and personal care	1 mild		
		2 moderate		
		3 severe		
SIB	Global cognition	0-100 points (<63 severe impairment)	Patient	Allows to evaluate very severe patients
ADCS-ADL	ADL	0-54 points	Caregiver	Allows to evaluate severe to very severe patients
	19 ADL of moderate to severe dementia			
FAST	Functionality	1-7	Health professional	Stages 6-7: divided into incapacity progression

## COGNITIVE EVALUATION

Cognitive evaluation in advanced dementia is relevant to measure pharmacologic and non-pharmacologic management and interventions. MMSE is the most frequently used instrument in brief cognitive evaluation in dementia. However, for moderate-late stages, we could observe a floor-effect, with few modifications over time below 10 points. At this point, we must consider other tests. The GDS described above could be an instrument for cognitive evaluation but severe-MMSE (SMMSE) or Severe Impairment Battery (SIB)^15^ ^),(^ ^16^ ^)^ could offer more detailed and objective assessments.

The SIB, consisting of 40 questions, evaluates nine areas of cognition: social interaction, memory, orientation, language, attention, praxis, visuospatial ability, construction, and orientation to name. Scores vary from 0 to 100.^15^

SMMSE is a brief test requiring minimal training and no special materials, but educational attainment influences it. Its scores vary from 0 to 30 and its items are divided into autobiographical knowledge, executive function, language, verbal fluency, and spelling^16^ ^),(^ ^17^ (Table 1).

If only one physician monitors cognitive status, they may ask the same questions to observe the progression of deterioration, such as age, date of birth, names of children or spouse, food or soccer team preferences.

## TREATMENT OF BEHAVIOR AND THE PSYCHOLOGICAL SYMPTOMS OF DEMENTIA

Behavior and psychological symptoms of dementia (BPSD) comprehends the neuropsychiatric manifestations besides cognition, affecting between 60 and 90% of people with dementia^18^. BPSD includes a wide spectrum of conditions: apathy, depression, anxiety, sleep disorders, psychosis, agitation, aggression, wandering and motor manifestations, disinhibition, and many others. Generally, BPSD prevalence increases with disease severity although epidemiologic studies in this topic mostly include small sample sizes, especially among those with AD^18^ ^),(^ ^19^. Moreover, severe forms of BPSD like delusions, hallucinations, agitation, aggression, and aberrant motor conditions are more common in moderate and severe dementia^20^. Multiple adverse outcomes have been associated with BPSD, such as cognitive and functional impairment, caregiver burnout, nursing home placement, and mortality^21^ ^)-(^ ^25^. However, research has scarcely explored outcomes in severe forms of dementia.

The occurrence and maintenance of BPSD relies on three factors, namely patients (e.g., hunger, pain, and acute medical conditions), caregivers (e.g., stress, lack of politeness, and communication skills), and the environment (e.g., under/overstimulation and lack of routine and activities). Consequently, it is rational and evidence-based that the first treatment step against BPSD includes non-pharmacological measures^26^. A systematic review and meta-analysis points to an effect size at least equivalent to those of psychotropic drugs, and it is safer^26^. However, in clinical practice, non-pharmacological measures are yet to be fully implemented due to several economic and cultural reasons. The next session will better explore this specific topic. Clinical practice recommends the observation of BPSD symptoms at different hours, with varying caregivers, and multiple environments. Moreover, research has also suggested using BPSD measurement instruments, such as the Neuropsychiatric Inventory (including the clinician version or NPI-C) and the BEHAVE-AD, due to multiple concurrent and sometimes complex symptoms.

After non-pharmacological measures or concomitantly to them, pharmacological treatment is possible and common due to safety reasons or BPSD severity. In general, response rates to different classes of medications are heterogenous and of small therapeutic effect. The overall evidence points to a small number of psychotropic drugs which reasonably improve BPSD. We recommend that these drugs be prescribed at low doses and that clinicians avoid, if possible, polypharmacotherapy. Table 2 summarizes our expert consensus-based recommendation for the use of psychotropic medication in BPSD associated with AD.

**Table 2:** Consensus-based recommendation for the use of psychotropic medication in specific behavior and psychological symptoms of dementia (BPSD) associated with severe Alzheimer’s dementia.

BPSD		Suggested dose range*	Side effects
Agitation or aggression	Citalopram**	10-20mg/day, single dose	nausea, diarrhea, headache, increased risk of falls
	Sertraline	50-100mg/day, single dose	
	Trazodone***	25-100mg/day, single/partial doses	
	Risperidone	0.25-1mg/day, single dose	extrapyramidal side effects, weight gain, metabolic abnormalities, hyperprolactinemia
Psychosis	Risperidone	1-3mg/day, single dose	extrapyramidal side effects, weight gain, metabolic abnormalities, hyperprolactinemia
	Quetiapine	100-200mg/day, single/ partial doses	drowsiness, weight gain, metabolic abnormalities
	Aripiprazole	10-30mg/day, single dose	nausea, weight gain, headache, somnolence, akathisia

*Doses are recommended based on clinical trials and personal experience, considering the pharmacological properties of these agents; **May consider escitalopram due to its greater safety and better cognitive profile. It is also more commonly prescribed in Brazil than citalopram; ***Although it has less evidence than the other agents, this drug shows a good balance between safety and effectiveness in clinical practice. Extended-release formulation is better during the day to avoid somnolence.

Treating cognitive impairment with cholinesterase inhibitors and memantine shows slight improvement in early and moderate stages of AD, according to a recent systematic review and meta-analysis^27^. Effects are small and benefits to which family members and caregivers referred may amount to a placebo effect. The benefits of these drugs for severe dementia are questionable but may exist and thus, clinicians must weigh the decision of maintaining or ceasing such treatment^27^. In moderate to severe AD, low to insufficient evidence suggested that cholinesterase inhibitors and add-on memantine inconsistently improved cognition and global clinical impression, compared to placebo. However, the evidence is questionable and deserves further exploration in clinical trials. Cholinesterase inhibitors and memantine also slightly improve BPSD, especially in the early and moderate stages of AD and include apathy, anxiety, and depressive symptoms. In total, three systematic reviews with meta-analysis^28^ ^)-(^ ^30^ have summarized this evidence. In general, attending physicians may consider prescribing and maximizing both cholinesterase inhibitors and memantine to improve cognitive function and BPSD before considering other psychotropic medication. Nonetheless, we emphasize that evidence is insufficient to decide in favor or against this position.

Citalopram and risperidone have produced favorable evidence for agitation in AD. Citalopram improved moderate agitation, if administered between 10-30mg a day^31^. Other antidepressant agents, such as sertraline and trazodone, improved agitation^32^. However, we must stress their potential limitations as one review included fewer patients with severe AD, still showing cognitive performance and a prolonged QT interval in patients receiving citalopram 30mg^31^. Ongoing S-CitAD, which evaluates escitalopram for agitation, may show greater safety and a better cognitive profile. Risperidone, mostly at low doses (0.5-1mg), and selective serotonin reuptake inhibitors (SSRI), as a class, alleviated agitation in patients with dementia, according to a systematic review and meta-analysis^32^ ^)-(^ ^34^.

The evidence for the use of antipsychotics for BPSD is limited even for agitation and aggression. Data from a systematic review showed that aripiprazole was the safest and most effective antipsychotic versus placebo, and it was associated with improved outcomes on the NPI, the Brief Psychiatric Rating Scale (BPRS), and the Cohen-Mansfield Agitation Inventory (CMAI) ^(^ ^35^. Quetiapine improved outcomes on the BPRS, and risperidone was associated with improved outcomes on the CMAI. Differences between atypical antipsychotics were insignificant for effectiveness, death, or cardiovascular adverse events^35^.

Apathy is a common BPSD, especially in early-stage dementia. The literature reports pooled evidence (small sample size from three studies) favoring the use of methylphenidate to reduce this symptom in the Apathy Evaluation Scale^36^. However, apathy is part of the phenotype of more severe dementia, and the overall cognitive decline summed with other bothersome BPSD in this stage results in a questionable pharmacological treatment of apathy.

A systematic review and meta-analysis^37^ ^)^ was unable to recommend the use of antidepressants to treat depression in BPSD based on its lack of efficacy in respond to or reduce depressive symptomatology. Benzodiazepines, anticonvulsants, and cannabidiols are also ineffective in pharmacologically controlling BPSD due to their lack of efficiency or adverse reactions, including cognitive decline. Finally, dextromethorphan/quinidine and prazosin showed evidence of improving agitation in AD^38^ ^),(^ ^39^ and pimavanserin reduced psychosis in AD^40^. However, these three agents are unavailable in Brazil.

Box 2 summarizes our overall treatment recommendation for cognitive dysfunction and BPSD. In conclusion, taken together, current evidence shows low certainty for prescribing cholinesterase inhibitors and memantine in severe dementia. The use of agents such as SSRIs, risperidone, and aripiprazole for agitation, aggression, and psychosis also showed a small or uncertain effect when we consider severe dementia. Non-pharmacological measures, including activities of daily living and care routines, proper feeding, pain control, music therapy, physical therapies, and caregiver education and support, seem to be safer and more effective^41^. The use of pharmacological agents should consist of single agents, in small doses, and for a short period to control the targeted BPSD (Table 2).

**Box 2:** Overall recommendation for the non-pharmacological and pharmacological treatment of cognitive dysfunction and behavior and psychological symptoms of dementia (BPSD) in severe Alzheimer’s dementia.

1. Consider withdrawing cholinesterase inhibitors and memantine in severe dementia in the absence of clear benefits to cognition or BPSD
2. Education and support for caregivers
3. Well established routine of daily care and activities
4. If possible, consider music therapy and any form of physical activity
5. Investigate causes for cognitive fluctuations or BPSD
6. Consider pain control before prescribing psychotropic agents
7. If agitated or aggressive, consider citalopram, sertraline or trazodone
8. If agitation or aggression persists with antidepressants, consider antipsychotics such as risperidone, aripiprazole or quetiapine
9. Always reevaluate withdrawing psychotropics for BPSD after symptom control

## NON-PHARMACOLOGICAL TREATMENT

### Nonverbal communication

*“Perhaps it’s the most important part of caring. Because it leads to reflection, because it provides the manifestation of human nature, present in each of the professionals involved with care. It is the deepest empathy and the need to show affection, affection and, above all, respect for the other. Holding the hands of someone who may have their mind lost, in another dimension perhaps, gently touching the thin skin of an already weakened body, looking deeply into their eyes and letting the feelings present in this exchange of glances lead to the understanding of what is needed to do. It is the meeting of the human and bioethics, a feeling of lightness and of certainty that the task is being carried out with respect and with the dignity that every human being deserves.” (Ceres Ferretti)*

The literature is very rich in papers discussing non-pharmacological approaches to psychological and behavioral symptoms in dementia - BPSD, which arise with the evolution of different dementia syndromes^42^ ^),(^ ^43^. However, unfortunately, the literature still lacks studies discussing the possibilities and effectiveness of these approaches in severe dementia, or even the difficulty of conceptualizing and adequately treating people with it^44^ ^)-(^ ^46^. Some behaviors are more prevalent in the initial phase of the disease, others in its intermediate phase and some in its severe phase. Its last phase signals a possible combination of factors intrinsic to patients (comorbidities) and caregivers (stress) and extrinsic (environmental) ones, which require multi and interdisciplinary assessment and conduct to make the best decision, case by case^47^.

Family doctors and multidisciplinary teams (previously trained by specialists) in primary health care units can theoretically recognize the needs and monitor patients with severe dementia who were discharged from the secondary care service. However, professionals working in this sector still face difficulties, related to their knowledge to perform diagnoses and administer pharmacological and non-pharmacological treatment, especially in mild and severe AD phases^48^.

Due to the problems in the health, economic, and social sectors in Brazil and the need for biopsychosocial support (especially during this serious health crisis^49^, reference and assistance centers have created projects which aim to minimize the direct and indirect social costs of PWD caregivers^50^ ^),(^ ^51^. These projects are in line with most guidelines from international consensus meetings of associations^52^ ^)-(^ ^57^, universities^58^ ^),(^ ^59^, and scientific committees^57^ ^),(^ ^60^.

All expert consensuses discuss the need for psychoeducational programs and suggest recommendations which contribute to the education of caregivers about systematized and appropriate models for different behavioral disorders. The great gap, however, is still the approach to the severe phase of AD. Knowing the causes of SCPD in this phase is the objective, and solving them, or better yet, preventing them is the goal^61^. The starting point is understanding possible social and more individual family barriers to the construction and practice of these models, those aimed at reducing these behaviors and, consequently, the comfort and quality of life of patients with severe dementia and their caregivers^62^.

### Assessment Models

In Brazil, the nursing model in dementia care includes all stages of disease evolution^63^. Research has shown that the protocol is useful in its main objective, which is to assess patients and their caregivers. Composed of two stages, patients and caregivers focus on patient-centered care models, in combination with the caregiver-centered care model^64^.

**Figure 1:**
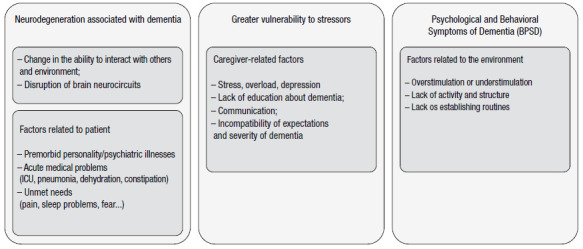
Depiction of a conceptual model for different interactions resulting in BPSD. Adapted from Kales (2015) ^(^ ^58^.

Kales (2015) ^(^ ^58^ offers a review with an interesting conceptual model of factors which require evaluation, leading to the reflection on the union of disciplines combining different areas of knowledge such as neurology, geriatrics, psychiatry, and gerontology in a multidisciplinary view (Figure 1). The same review shows “DICE - Describe, Investigate, Create, and Evaluate,” proposed by the University of Michigan in partnership with the Johns Hopkins Alzheimer’s Disease Research Center and the Center for Innovative Care in Aging with guidelines to identify needs for the practice of care^58^.

As mentioned above, clinical practice still lacks a consensus on the definition of severe dementia, sometimes confused with moderate dementia. However, studies agree on possible causes that give rise to common behaviors, such as agitation, apathy, hallucinations, delusions, “unmotivated” crying, fear, anxiety, contracted body, and resistance to care, among others^58^ ^),(^ ^62^.

Several studies report that most factors responsible for SPCD in severe dementia originate from patients’ comorbidities, caregivers’ lack of knowledge and stress, and the environment. The inability to verbally communicate can lead to the behaviors in the same way that the rapid recognition of any change in the pattern of patients’ usual behavior can prevent progressing of the SPCD and interfering with the quality of life of both patients and caregivers (Table 3) ^(^ ^58^ ^),(^ ^65^.

**Table 3:** Recognition and Management Recommendations for Psychological and Behavioral Symptoms in Severe Dementia. Adapted of Kales (2015) ^58^.

In the patient	In the caregiver	In the environment	Recommendations
Pain			Caregivers’ training;
			Health education;
	Lack of knowledge	Poor positioning	Identification and elimination of causal factors in patients and the environment;
	Health education	Excessive noise	Promotion of physical comfort;
	Lack of education in dementia	Abrupt movements	Information to the responsible physician and members of the health team;
			Elimination of excessive noise;
			Gentle patient mobilization.
Urinary infection		Inadequate hygiene	Caregivers’ training;
			Health education;
	Lack of knowledge		Optimization of fractional hydration (1.5-2L/day) or at medical discretion;
	Stress		Double incontinence;
			Hygiene, body, and environmental measures.
Respiratory Infection	Lack of knowledge	Dark, closed or dirty environment	Caregivers’ training;
		Cold temperature	Preventive measures;
			Passive exercises.
Constipation	Lack of knowledge	Adequate bathroom hygiene, security, and privacy	Training in hygiene measures and health education to caregivers;
			Medical and nutritional approach;
			Fractioned hydration.
Refusal of care	Inadequate communication and conducts	Environment adaptation and care	Training of caregivers and health education;
			Caregivers must be careful with all stimulus and approaches.
Sleep disturbances	Lack of recognition concerning pain, hunger, coldness, heat, mobilization in bed, and previous sleep problems	Observation	Training and health education;
		Change in patients’ behavioral patterns	Adequacy of auditory environmental stimuli;
			Medical evaluation.

* All caregivers must be trained in all activities proposed by the health team to offer their consent and understand the importance of each proposed intervention. Elaborated by Ferretti (2021).

### Comorbidities in advanced dementia

In general, older adults, and especially those with dementia, face exclusion from clinical trials on pharmacological interventions and the medical literature is scarce in good-quality data regarding the advanced stages of dementia since the focus of guidelines and randomized, controlled studies and intervention are aimed at premorbid, very mild, and mild stages of dementias^66^ ^),(^ ^67^.

Although research has suggested the possibility of AD patients being healthier^68^, current evidence points to the opposite: people with dementia (PWD) show a significantly higher prevalence and medication use^69^ ^)-(^ ^73^.

Clinical diseases impact quality of life (by, for example, worsening mobility) and patient survival regardless of dementia, both in institutionalized individuals and those who remain in the community, increasing the number of searches for emergency rooms, hospitalizations, hospital costs, and health expenditures^69^ ^),(^ ^70^.

Older adults with dementia are three times more likely to have four or more concurrent chronic diseases and annual expenses, up to 3.3 times higher than in older adults without this disease. These comorbidities impact survival throughout patients’ later years^74^ ^),(^ ^75^ (Table 4).

**Table 4:** Conditions associated with higher mortality among subjects.

Number of diagnoses
Male
Age
Lung infections
Parkinsonism
Previous Stroke
Atrial fibrillation
Malignancy

As for the severity of dementia, results pointed to a significantly lower two-year survival rate among most dependent patients, regardless of their number of comorbidities. We find a positive correlation between number of comorbidities and dementia severity independently but not the impact of the interaction between them; the more advanced phases compete with higher health expenditures^73^.

Schubert et al. highlight the possibility that the lesser search for diagnoses and treating comorbidities in PWD on an outpatient basis may be one of the conditions responsible for its aggravation, showing the need to search for advanced care units, which could be avoided under a less nihilistic outlook^72^ ^),(^ ^76^ ^),(^ ^77^(Box 3).

**Box 3:** Chronic conditions most frequently associated in PWD.

• Heart failure
• Hypertension
• Diabetes mellitus
• Neoplasms
• Kidney failure
• Cerebral and coronary artery diseases
• Atrial fibrillation
• Lung diseases

### Dementia in comorbidities

As previously mentioned, dementia directly impacts the survival of individuals regardless of the presence of comorbidities^75^. Dementia decreases survival after acute myocardial infarction, with greater renal decompensation, higher rate of acute lung edema, showing the delay in hospitalization and the lesser prescription of antiplatelet agents and beta-blockers at hospital discharge^77^. Dementia also predicted higher mortality in patients with heart failure^78^.

The frequent coexistence between Advanced Dementia and Frailty syndrome could be one of the ways to explain, at least partially, the worse prognosis of comorbidities among PWD^79^. As it is known, although the definition of frailty is still a matter of debate in the current medical literature, frail individuals, especially the elderly, have less functional reserve of physiological organs and systems and less capacity to face challenges and organic overload, having greater difficulty in maintaining homeostasis when faced with acute illnesses, interventions of any kind, diagnostic or therapeutic^80^.

The management of comorbidities in dementia, on the other hand, faces several difficulties regardless of what has been exposed so far: starting with making a diagnosis in these individuals, who, due to the progressive impairment of language, memory, and criticism the efficiency in reporting symptoms and complaints is lower, less valued or commented upon and dependent on a third party not always attentive or accurate (the caregiver). Adherence to medication and non-pharmacological guidelines is also erratic and variable, by patients and caregivers^69^ ^),(^ ^81^. Moreover, once undiagnosed and addressed with an adequate care plan at an outpatient level, such illnesses become more complicated and patients end up being taken to emergency rooms, wards, and Intensive Care Units, often with treatments that are disproportionate to their condition. advanced disease^69^ ^),(^ ^81^.

Thus, often, despite having a high number of comorbidities, patients with dementia are less likely to have their health problems diagnosed and treated, including those that may directly impact their functionality, such as auditory and visual sensory alterations^71^. Often, by the simple mention of the diagnosis of dementia, especially if advanced (albeit moderately), such individuals are deprived of beneficial proportional treatments due to discrimination since the diagnosis suggests that these patients would fail to obtain the benefit of a certain procedure, even though they might live long enough to experience the complications of the disease, thus sometimes imposing suffering which could have been avoided^72^ ^),(^ ^82^.

The reduction in survival expectancy should limit the administration of treatments unable to improve patients’ symptoms or quality of life. Thus, clinical practice should rethink primary preventive treatments in light of the prognosis to avoid the unnecessary use of medications and polypharmacy and control diseases such as hypertension and diabetes mellitus, and dyslipidemia. Moreover, other secondary prevention measures must comply with current guidelines indicating less strict goals for older adults with advanced ages and those with lower survival expectations^83^.

### Comorbidities of dementia

The more advanced stages of dementias, especially in its terminal stages, induce the increase of morbid conditions such as lung infections due to bronchial aspiration or other causes, immobility, dysphagia, urinary tract and skin infections, pressure injuries, falls, malnutrition, and various dental problems^84^.

Maintaining patients’ dignity, regardless of their cognitive status, treating and preventing reversible complications (considering individual and family values), and establishing, as early as possible, a plan to consistently care for these patients with the best technical-scientific knowledge, are the pillars to treat individuals with any diagnosis, including dementia, whatever their etiology, patients’ socioeconomic condition or stage^81^ ^),(^ ^82^ ^),(^ ^84^ ^),(^ ^85^.

### Cancer and dementia

Both cancer and dementia have increased prevalence with age and lead to very complex health care needs and worse outcomes in people suffering from these diseases than those without these comorbidities^86^. Estimates of the prevalence of the association between the two diseases vary in the literature. An estimate suggests that 7.5% of individuals over 75 years old live with both diagnoses^86^ ^),(^ ^87^. People living with this dual diagnosis are less likely to receive screening, staging, curative treatment, and adequate pain management than cancer patients without dementia^86^ ^),(87^. Moreover, they have late diagnoses, a lower survival rate after it, and a greater number of comorbidities than those living only with cancer or dementia^87^.

Oncology services poorly identify dementia and its patients often receive limited therapeutic options. Oncology teams feel insecure about managing these patients since dementia carries more complex decision-making. Finally, studies highlight the important role families play in promoting greater success in treating and managing cancer in patients with dementia^86^.

Cancer diagnosis and treatment for patients in very advanced stages of dementia refrain from administering screening and very invasive measures. Even so, clinical practice must establish a care plan to ensure quality at the end of life for people who have aged with these two diseases: pain management, prevention of avoidable complications, and family support.

### Approach to pain in advanced dementia

Older adults with dementia shown a 32-53% estimated prevalence of pain, higher among those with diseases known to cause pain, such as osteoarthritis, fractures, peripheral arterial disease, and cancer. This prevalence can reach 83%¨in those living in ILPIs. No study has shown that dementia affects pain sensitivity; rather, what it alters is individuals’ ability to report what they are feeling^88^.

Research considers self-reports as the gold standard in pain diagnosis. Patients with dementia show an unacceptable underdiagnosis and undertreated pain^88^ ^)-(^ ^90^. Among institutionalized older adults, 25% of those who daily complained of pain had received no analgesics. Among those with hip fractures and dementia, a student found that the prescribed opioid was a third of the normal dose and, therefore, insufficient ^(^ ^89^ ^),(^ ^91^.

Untreated pain manifests itself via secondary symptoms in patients with dementia, such as sleep disturbances, agitation, depression, weight loss, and decreased mobility^90^. Studies have shown that analgesics better control agitation in PWD and pain than neuroleptics, especially in moderate-advanced dementia. They also reduce aggressiveness and pain without worsening patients’ cognition ^91^ ^),(^ ^92^.

To optimize pain assessment in PWD and, therefore, its identification, a study developed a specific pain scale for severely demented patients, later validated for Brazilian Portuguese^90^. The instrument (PAINAD) evaluates five items: breathing; negative vocalizations; facial expressions; body language; and comfortability, observing the patient for five minutes in different daily situations: during rest, in pleasurable activities and moments of care; and 30 minutes after analgesic medication. Multidisciplinary teams can use it after training without impeding its complexity.

Thus, evaluating and treating pain in patients with advanced dementia is a challenge that clinical practice must face and, whenever suspected due to its manifestations, approach and treat it.

### Oral health

People with dementia have the same oral problems as the general population. Good oral health positively influences individuals’ overall health, dignity, self-esteem, social integration, and nutrition. Studies show the effects of oral problems in patients with dementia with difficulty chewing due to missing dental elements and consequent refusal to eat, behavioral changes (such as withdrawal and aggressiveness due to pain - as previously mentioned), and other changes. The nature of dementia and its severity, social functioning, behavioral aspects, adherence to oral cavity care, and caregivers’ ability of caregivers to replace them in this care may compromise the conditions for maintaining oral health^93^ ^)^ (Table 5).

**Table 5:** The main changes seen in the oral cavity of patients with dementia are:

• Poor hygiene
• Gingivitis with accumulation of bacterial plaque, calculi, and bleeding
• Caries
• Fractures with remaining roots and eventual infection
• Ulcers, gingival hyperplasia, and lack of taste due to psychotropic drugs often used to control symptoms in these patients.

Overall health and comfort are closely linked to oral health in the terminal stages of neurodegenerative diseases^94^ (Table 6). Oral diseases worsen general comfort, cause pain, affect cognition and behavior, and alter quality and life expectancy of people with dementia. The risk of aspiration pneumonia increases in the presence of oral factors such as poor hygiene, meager coronal and cervical teeth, periodontal disease, and the presence of microbes in saliva, whereas a clean and healthy oral cavity significantly reduces its occurrence^95^ ^),(^ ^96^.

**Table 6:** Factors which negatively influence oral health^93^.

• The severity of dementia
• Previous dental history - care and diseases
• Ability to receive / consent to oral hygiene care by caregivers or dental teams
• Knowledge of patients or their caregivers about the importance of oral health
• Lack of patient/caregiver motivation
• Impacts of medications on the oral cavity (xerostomia)
• Lack of information on how to access teams
• Degree of knowledge/training of oral health teams regarding dementia and aging
• Teams’ failure to develop strategies and long-term care plans.
• Scarcity of appropriate care facilities for small/medium dental surgeries, day centers, and at home.

Caregivers play a central role in the oral health of patients with advanced dementia. The quality of hygiene care and the perception of problems which may arise depend almost exclusively on them. Studies have shown a poor understanding of the importance of oral health and its subsequent problems, such as patients’ (often aggressive) resistance further complicate the adherence of caregivers to these demands. Education, motivation, and the offer of strategies was associated with improved oral hygiene in patients with severe dementia^97^. Regular odonatological visits, caregivers’ education and access to care facilities in day centers, outpatient clinics, and home services are strategies which can ensure better oral health for these patients, benefitting their global health and quality of life increasing their expectations of survival.

## NUTRITIONAL ISSUES IN ADVANCED DEMENTIA

### Undernutrition in advanced dementia

As dementia advances into a severe stage, feeding difficulties become more common and bring about important problems such as undernutrition and weight loss, which are associated with more rapidly progressing cognitive impairment and increased mortality^98^. Therefore, all PWD should receive routine assessments of their nutritional problems. Several instruments can identify whether patients are undernourished, such as the Mini Nutritional Assessment (MNA) and the Malnutrition Universal Screening Tool (MUST)^99^ ^),(^ ^100^. An unstructured evaluation is, however, also a useful option, particularly under limited time, common in Brazilian primary care consultations. These evaluations should include anthropometric measurements (weight, height, and body mass index) and questions about food and fluid intake, dietary habits, and adversative feeding behaviors. Laboratory tests are also helpful to better evaluate patients’ nutritional status, including full blood count, electrolytes, B12, urea, creatinine, glucose, albumin, and ferritin.

Individuals with advanced dementia may show adversative feeding behaviors that can importantly contribute to undernutrition, such as refusal to eat, wandering, and agitation^101^. Research has little evidence that interventions to modify mealtime environments can improve food and fluid intake for these patients^102^ ^),(^ ^103^. However, given their favorable risk-benefit and the possibility of improving patients’ quality of life, employing such measures is reasonable^104^. Box 4 describes our suggested interventions.

**Box 4:** Measures to improve mealtime environment in advanced dementia.

Refrain from rushing; good interaction between patients and caregivers increases food intake
Offer food patients like
If possible, the same caregiver should always be responsible for assisting patient’s meals
Try to set a pleasant, homelike environment with improved lighting and familiar music
Although it is useful to maintain a routine, it is advisable to wait until patients are calm before offering them food and fluids.
Use high-contrast colored tableware
Consider offering regular snacks and small meals

Oral Nutritional Supplements (ONS) can increase macronutrient intake and avoid undernutrition in older adults, including persons with advanced dementia. As a meta-analysis shows, ONS can increase weight in persons with dementia, showing few gastrointestinal side effects. Note, however, that the literature lacks enough evidence to aclaimffirm that ONS decrease mortality or cognitive deterioration among persons with dementia^105^.

### Dysphagia in advanced dementia

Oropharyngeal dysphagia is a serious and common problem in advanced dementia, as it may be an important cause of undernutrition and increase the risk of respiratory infections and death^106^. Early stages of dementia may show subtle aspirations and even go unnoticed by patients or caregivers^107^. Therefore, speech and language therapists should take part in managing PWD as early as possible, even in the absence of swallowing complaints. The most important instrument for diagnosing dysphagia on a day-to-day basis is the Clinical Swallow Evaluation (CSE). it includes both questionnaires on swallowing problems and a motor and sensory examination of all oral structures involved in bolus formation^108^ ^),(^ ^109^. More specific situations (e.g., to diagnose more accurately aspiration) may require employing an instrumental assessment, such as videofluoroscopic swallow studies (VFSS) and fiberoptic endoscopic evaluation of swallowing (FEES)^108^ ^)-(^ ^110^.

The use of thickeners to change the consistency of fluids offered to patients with advanced dementia can help to mitigate the consequences of dysphagia. In 2018, a meta-analysis concluded that thickening the fluids offered to patients with advanced dementia may have an immediate positive effect on swallowing and may decrease the three-month incidence of pneumonia^111^. It noted, however, that long-term benefits are uncertain due to scarce evidence^111^. Research also lacks enough evidence to recommend a specific thickness (i.e., nectar- or honey-thick) over the other^112^. Adopting a chin-down posture while drinking liquids is another useful intervention in dysphagia and may be as effective as fluid thickening to decrease the incidence of pneumonia, especially in individuals with milder dysphagia^112^ ^),(^ ^113^.

### Tube feeding in advanced dementia

Using or withholding tube feeding remains one of the most controversial topics in the management of advanced dementia. Caregivers often misinterpret the recommendation to not insert a percutaneous endoscopic gastrostomy (PEG) as a recommendation to withdraw all types of care, which might compromise ongoing treatment and even harm the confidence in healthcare providers. Of note, such recommendations are based mostly on observational studies and not on randomized controlled trials, as these are unavailable due to ethical reasons^114^ ^),(^ ^115^.

Most of the available evidence suggest that tube feeding fails to benefit patients with advanced dementia and may even harm them. The use of PEG tubes seems to fail to improve mortality in individuals with advanced dementia^116^ ^)-(^ ^118^, may lead to higher levels of discomfort^119^, and its complications are responsible for almost half of all emergency department visits among patients with advanced dementia^120^. Tube feeding also seems to increase the risk of pressure ulcers and are unhelpful to heal existing pressure ulcers^121^ ^),(^ ^122^. Finally, enteral feeding with a PEG tube fails to decrease caregivers’ burden^121^ and raise a perception of better end-of-life care among relatives of individuals with advanced dementia^123^. Current evidence still remains unclear, however, on whether such adverse outcomes are related to the late use of PEG tubes and if patients in less severe stages of advanced dementia could benefit from it ^(^ ^117^ ^),(^ ^124^.

The decision to withhold tube feeding in advanced dementia is ethical and based on current scientific evidence but should not be viewed as a one-size-fits-all way to approach the matter. The discussion should include families and, whenever possible, patients, and consider their social, cultural, and religious values. In other words, the decision to use or withhold use PEG tube feeding must be individualized. Whenever tube feeding is contraindicated, assisted oral feeding is encouraged. The concept of “comfort feeding only”^125^ ^)^ is useful in such situations: it is based on feeding with comfort as its main goal, i.e., focusing on satisfaction and stopping whenever feeding is distressing.

## END-OF-LIFE CARE IN DEMENTIA

People with advanced dementia may possibly include a great part of the patients living with dementia in Brazil. However, trustful estimates are still impossible due to the lack of epidemiological data and the profile of this population. Advanced dementia patients show a low income, associated with a high disease burden profile, often lacking the assistance of formal caregivers^126^. Delivery of care to PWD is essentially provided by family members in Brazil^127^, unlike European countries with a public health system, such as the United Kingdom^128^. Thus, unique characteristics of the Brazilian people and its continental-size health system demand adapted recommendations for appropriate end-of-life care of people with dementia.

End-of-life care, or palliative care, is a therapeutic strategy to maintain a person’s quality of life by relieving discomfort or stress in a life-limiting condition. This subtopic details a few steps to aid general practitioners and primary care physicians: (1) accurately identify the moment of defining end-of-life care, (2) plan next steps with patients and their family members, and (3) provide mental and physical assistance to patients with terminal illness.

### Identifying the moment of end-of-life caring

Recognizing individuals with dementia in advanced disease stage is a cornerstone to provide adequate strategies. In Brazil, almost 80% of individuals with dementia are still undiagnosed^129^. A sensitive, accurate feeling that forgetfulness may not be solely associated with the aging process is the first step to provide proper care of patients with severe dementia. Ideally, the moment of discussing palliative care is as early as dementia is diagnosed, focusing on patients’ wishes. Family members are essentially involved in deliberating decisions when individuals no longer may take complex decisions^130^.

Primary care physicians should have a high suspicion of dementia even in patients without cognitive complaints^131^. Once they identify the cognitive decline, the next step is defining its stage. Clinical features vary in severe stage dementia, including mutism, impaired *per os*, and severe gait disturbances*.* Recurrent hospital admissions or demanding frequent medical assistance may be early signs of end-of-life disease, especially involving a recent diagnosis of cancer, heart failure or chronic obstructive pulmonary disease. Primary healthcare professionals should also pay attention to mental status of patients in home care appointments, as a great number of individuals were home assisted, lacking appropriate follow-up^132^.

### Planning the approach to patients and their families

The Brazilian public healthcare system has a major infra-structure to provide an interdisciplinary approach to families with patients in the end-of-life dementia stage. Primary care facilities are strategically located close to the local community, which increases confidence and access to healthcare. However, most public facilities lack psychologists, physical therapists, speech therapists or social workers.

Once the diagnosis of end-of-life dementia is established, the next priority is to discuss an interdisciplinary approach to provide optimal assistance to loved ones. Only a minority of older adults have access to long-term care institutions^133^. Although interdisciplinary care is essential to mitigate end-of-life discomforts involving different healthcare professionals, Clinical practice should implement a broad care for those with access to private physical, occupational, psychological, and speech therapy.

For most patients with limited access to these services, we must discuss a few points. Firstly, family members must immediately increase awareness of end-of-life care. They should raise a community spirit, reach local policies, and increase their voice on a nationwide level. Philanthropic institutions such as “Associação Brasileira de Alzheimer” (ABraz) may provide information about local hospices or low-cost services, though they often have limited regional actions. All levels of care should also address spiritual needs, as sect leaders should aid planning at the time of diagnosis^134^.

### Providing mental and physical assistance for patients (and caregivers)

Assisting people with terminal dementia should consider several levels of care^135^. The relatively weak healthcare structure for patients with terminal diseases compels families to establish a plan of care for persons with dementia. The whole primary care team should perform timely planning discussions, including stratifying care demanded by both patients (when cognitively able to take decisions) and caregivers^136^.

Ideally, they shall create a plan of care whenever patients receive a diagnosis of end-of-life dementia. They must assess patients’ understanding and judgement to evaluate their wishes; if impossible, this decision should involve their families or caregivers. Patients with end-of-life dementia benefit from care levels divided into five stages, ranging from critical care including invasive procedures to supportive care and comfort measures only^137^ ^),(^ ^138^. Care level must consider patients’ wishes regarding invasive procedures in general, including orotracheal tube, central venous catheter, cardiopulmonary resuscitation, and ICU admission (Table 7). Caring should be individualized and performed together with families.

**Table 7:** Medical Orders for Scope of Treatment (MOST), in Portuguese, adapted to patients with end-of-life dementia stage. Staging must consider wishes to admission in a critical care unit, orotracheal intubation and cardiac resuscitation.

Plan demanded by the patient	Medical record	Plan of care	Level of care
I want my life to be preserved using all measures of care available, when indicated	**Full intensive care**	ICU admission, including orotracheal intubation and CPR	**Critical care:**
I want my life to be preserved, except when my heart stops. I accept ICU admission and all measures, including intubation, except CPR if my heart stops.	**Intensive care without CPR**	ICU admission. Excluding: Defibrillation and CPR	- Attempt to support life with artificial measures in ICUs.
I want my life to be preserved, except when my heart stops. I accept ICU admission and all measures, except CPR if my heart stops or in case of intubation.	**Intensive care without orotracheal intubation or CPR**	ICU admission Non-invasive ventilation and high-flow O2 Excluding: Intubation, defibrillation, and CPR	- These orders in general are not used in case of natural end-of-life outcomes.
I want to be treated for reversible causes but without measures of artificial life support. If I show an irreversible condition, I want to receive the treatment available, except for invasive procedures (intubation or CPR). I want to receive this care in this ward.	**Advanced ward care**	Ward care, including treatment for reversible causes. Excluding: ICU admission, intubation or CPR	**Ward care:**
I understand death as a natural, expected event. I accept receiving care to decrease pain or other symptoms. I would like to receive this set of care whenever available.	**Basic ward care**	Exclusive symptoms management and comfort measures	- Support artificial measures will not be initiated if patients’ heart or breathing stops (i.e., intubation or CPR)

Education for caregivers and family members is a fundamental point that must be thoroughly discussed. Instructions such as available sources of information about end-of-life dementia, where to ask for help, when to ask for assistance (or be taken to the ER), and what is expected and what is not. Assistant physicians may provide adequate end-of-life care, including adequate management of pain, agitation/aggression, risk of aspiration, and need for a feeding tube, dyspnea, and pneumonia^139^.

Caregivers’ mental health must also be addressed. Since they usually are a family member, they often show a psychological distress associated with the caregivers’ burden^140^ ^),(^ ^141^. Caregiver assistance should focus on providing a better quality of life for the entire family. Avoiding psychological distress should be a priority, and we must suggest them to perform rotation shifts between caregivers (other family members or half-time support caregivers). Mental health hotlines have also been progressively implemented to assist those with psychological needs in Brazil^142^, especially after the COVID-19 pandemic^143^.

Thus, end-of-care dementia demands unique, individualized strategies which assistant physicians should recognize. Early identification, conjoint planning with families, and with level of care staging are cornerstones of adequate dementia care.

## ETHICAL AND LEGAL ASPECTS

Countless ethical and legal issues arise and evolve regarding the stage and severity of neurodegenerative diseases. These disorders compromise the psychological well-being and behavior of patients, impacting their quality of life, and physically and emotionally harming their families. Dementias generate loss of autonomy and inability to make decisions, pillars of medical ethics which support the management of diagnostic or therapeutic medical approaches^144^.

In its advanced stage, PWD are unable to care for themselves^145^. Advance care planning (ACP) involves a dynamic process based on a dialogue between individuals, close ones, and healthcare providers and concern future preferences about their medical treatment. Although referring to advanced dementia and contemplating favorable intentions, they are unable to predict all possible scenarios for the most appropriate decision-making. Patients have their own characteristics; diseases have different courses, and the future is unpredictable^146^.

Over the past few years, several countries have implemented laws regulating care for the dying. Without a doubt, the issue is controversial, especially regarding shortening patients’ life; colorful debates address euthanasia, though very timidly in Brazil. In patients with severe dementia, the situation becomes more complex due to patients’ inability to make decisions and report their feelings and suffering^147^.

The literature on severe dementia is significantly reduced, containing few studies with reliable indicators and guidelines directing healthcare providers in this moment of innumerable doubts and uncertainties. The lack of scientific evidence predisposes the prescription of aggressive therapies without concern for their insignificance, especially in intercurrent diseases, such as the lack of results showing the effectiveness of artificial nutrition and hydration. Thus, to avoid procedures incompatible with the dignity of the human life, we need to advance concepts such as which prognostic criteria best fit survival, how to assess suffering and quality of life in this special population, and for how long the medications available could prolong life^148^.

In this sense, evoking the Hippocratic maxim of “primum non nocere,” i.e., above all do no harm, is always an appropriate judgment^145^.

It is essential to reflect on the development of technologies that prolong life. Clinical practice may falsely perceive that this has been accompanied by an improvement in the quality of life. Living longer fails to necessarily mean patients have had an adequate quality of life, as the opposite is more often the case^149^. Therefore, when questioned, people claim that living with quality of life is more relevant than living longer but the measures adopted in many assistance services may be incompatible with this desire. Living without a minimum of quality is unacceptable for a considerable portion of the cognitively competent population.

In severe dementia, cardiopulmonary resuscitation and other life support measures may seem futile and should be carefully evaluated according to the patients’ previous wishes. Refusal to eat and dysphagia are late manifestations and uncomfortable ethical dilemmas for doctors who care for older adults. The decision to insert food via an alternative route is complex and dependent on a multidimensional understanding^144^.

In the end, our approach must be proportional to patients’ needs and based on bioethical principles. A bioethical perspective that studies life not only from a biological point of view but also from a biographical one, with the maintenance of life as a right rather than obligation^149^.

### Decision-making capacity

Ethically, screening and evaluating individuals for skills such as decision-making capacity (DMC) should be patient-centered and based on a functional assessment, rather than on expectations from an instrument or scale and its quantitative analyses. In dementia, substitute decision makers (SDM) are authorized to have frank conversations to predict and document patients’ desire prior to their disability. Even when a SDM is appointed to provide legally effective consent or refusal, this fails to render patients’ preferences ethically irrelevant^150^.

In accordance with the DMC issue, doctors must understand legal consequences, as they are responsible for patients and will always be asked, when necessary, to report information officially attesting the disease, as well as briefly describing patients’ conditions within expectations. One of these situations is the possibility of a guardianship procedure for individuals with dementia, depriving them of the legal capacity to make decisions and manage their assets. The Brazilian judicial determination of disability (a legal institution provided for in the legislation) finds only a small number of interdicted patients at an advanced stage of dementia. The main intention of the interdiction is to protect individuals with a legally significant disability. The Civil Procedure Code regulates these guardianship processes, “interdiction” in the Brazilian legal language. This means that Brazil faces a significant lack of legal responsibility in severe dementia, probably resulting in numerous inappropriate measures which fial to reflect patients’ real needs and interests^151^.

### Topics to deal with ethical dilemmas in the advanced stages of chronic diseases such as dementias

The concept called “Jonsen’s 4-topic” is a practical approach and structure used by many ethics committees to resolve clinical ethical dilemmas. It includes a categorization into four similarly weighted quadrants composed of information, facts, and descriptions (Box 5)^144^ ^),(^ ^145^ ^),(^ ^151^ ^),(^ ^152^.

**Box 5:** Bioethical topics.

Medical indication	Patient preference
benefits X harms/favoring patients	principle of autonomy/honor and respect the patients’ wishes
It refers to the practice of doing good and benefiting others against acts which may be harmful, helping health professionals choose available treatments and examining how each alternative raises the possibility of success and favors patients.	It applies the ethical principle of autonomy and examines patients’ previously expressed or assumed beliefs and preferences. It aims to honor and respect patients’ wishes as much as possible within acceptable limits. In severe dementia, it would be supported by an advance directive, with the need for a SDM as a family member or friend to help with decision-making. In the absence of advanced directives, it is essential to request the presence of an SDM. This enables caregivers to determine how patients would like to be cared for if they were able to decide, whatever it may be. It would involve their morals, hopes, aspirations, values, principles, and spirituality. Therefore, sensitivity of those responsible is paramount, which is practically the current rule in Brazil.
**Quality of life**	**Contextual features**
Dignity/safety and comfort	socio-economic factors/political and religious preferences
The various treatments available should provide a better quality of life (QOL). Dimensioning QOL is complex and unique for everyone, even with the help of family members’ judgment, care must be taken not to prolong life with suffering. Dignity, safety, and comfort must prevail.	This last quadrant refers to the exacerbation of ethical dilemmas due to socio-economic, political, cultural, and religious factors that frequently appear in advanced dementia and are reflected in care and decision-making.

In conclusion, the general approach to patients with advanced dementia is, as can be seen from the above, quite complex. The range of information, difficulties in diagnosis, peculiarities in treatments, and the mandatory inclusion of family members in decision-making impose extensive and frequent care to guarantee these individuals the best care based on the best evidence and, in the absence of such, the best experience and common sense available. To this end, we suggest that patients and their caregivers are evaluated very closely, at least every three or four months, to maintain their best possible functionality and maximum comfort, and that the following aspects receive attention. Respectful care, based on the best evidence, individual and family values, and the search to clarify any remaining doubts should guide the medical practice before patients and their loved ones.
